# 3D coaxial out-of-plane metallic antennas for filtering and multi-spectral imaging in the infrared range

**DOI:** 10.1038/srep28738

**Published:** 2016-06-27

**Authors:** Andrea Jacassi, Angelo Bozzola, Pierfrancesco Zilio, Francesco Tantussi, Francesco De Angelis

**Affiliations:** 1 Istituto Italiano di Tecnologia–via Morego, 30, I-16163 Genova, Italy; 2 Università degli Studi di Genova, via Balbi, 5, I-16126, Genova, Italy

## Abstract

We fabricated and investigated a new configuration of 3D coaxial metallic antennas working in the infrared which combines the strong lateral light scattering of vertical plasmonic structures with the selective spectral transmission of 2D arrays of coaxial apertures. The coaxial structures are fabricated with a top-down method based on a template of hollow 3D antennas. Each antenna has a multilayer radial structure consisting of dielectric and metallic materials not achievable in a 2D configuration. A planar metallic layer is inserted normally to the antennas. The outer dielectric shell of the antenna defines a nanometric gap between the horizontal plane and the vertical walls. Thanks to this aperture, light can tunnel to the other side of the plane, and be transmitted to the far field in a set of resonances. These are investigated with finite-elements electromagnetic calculations and with Fourier-transform infrared spectroscopy measurements. The spectral position of the resonances can be tuned by changing the lattice period and/or the antenna length. Thanks to the strong scattering provided by the 3D geometry, the transmission peaks possess a high signal-to-noise ratio even when the illuminated area is less than 2 × 2 times the operation wavelength. This opens new possibilities for multispectral imaging in the IR with wavelength-scale spatial resolution.

During the last decade the field of infrared (IR) plasmonics attracted a growing scientific interest^1^,^2^. The medium- and long-wavelength IR ranges extend from roughly 3 to 15 μm, and they are of particular interest for biological analysis^3^,^4^,^5^,^6^,^7^, sensing^8^,^9^,^10^, and security applications^11^,^12^,^13^. In fact, the spectral signatures of many biological compounds as well as the blackbody emission peaks of living beings, astronomical and mechanical objects all fall in this spectral range.

Nanocoaxial metallic structures represent an interesting family of systems with two key features. The first is wavelength selectivity which makes them appealing for IR filtering^14^,^15^, waveguiding^16^,^17^ and multi-spectral imaging^18^. The second is the presence of intense hot-spots of electromagnetic energy density. This feature can boost the development of emerging disciplines such as non-linear Optics^19^ at the nanoscale, magnetic hot-spots generation^20^, optical trapping^21^, and negative-index materials^22^.

In this work we focus on IR filtering and multi-spectral imaging from a general point of view. By multi-spectral imaging we mean any imaging technique where photons of different wavelengths are captured and analyzed separately. The impinging IR spectrum is divided into several bands of interest. In a CCD architecture, each pixel can be engineered to analyze a specific wavelength. In order to maximize the spatial resolution, it is important to minimize the size of the pixel down to the wavelength scale while preserving its wavelength selectivity. In this sense, the best commercial filters for spectral analysis in the IR are not well suited for such a downscale of the size. In fact, these are based on Fabry-Pérot cavities, where a layer of transparent dielectric material is sandwiched between two reflecting surfaces (either thin metallic layers or multilayer DBR stacks). Despite having very high transmission up to 95% and a narrow bandwidth of just a few tens of nanometers^23^,^24^,^25^,^26^, these are bulky optical components. The wavelength tuning requires changing the cavity length, which is impractical if micron-size pixels have to be densely packaged in a CCD array.

To overcome this limitation, several photonic^27^,^28^,^29^ and plasmonic^14^,^15^,^30^,^31^,^32^,^33^,^34^ filters have been proposed during the last decade. These are based on periodic arrangements of nanoscale apertures, and the wavelength tuning can be done by changing the lattice period of the array. The vast majority of the designs proposed in the literature are actually bi-dimensional, i.e. they are flat and do not extend in the out-of-plane dimension (z).

Here we show a new configuration of 3D metallic coaxial antennas for IR applications which combine the strong in-plane light scattering provided by the out-of-plane geometry with the selective spectral transmission of 2D arrays of coaxial apertures. Each antenna has a multilayer radial structure, with an outer dielectric shell that encapsulates a metallic hollow core. Along the vertical direction, a metal plane is inserted normally to the main axis of the antennas. Thanks to the 3D design, our antennas can scatter light in the lateral direction with greater efficiency compared to 2D arrays of annular apertures. The nanometric gap between the antenna and the metallic mid-plane allows the flow of light to the other side of the sample in a set of well-defined resonances. Their spectral position and bandwidth can be tuned by controlling the geometrical parameters of the structure. We demonstrate that the transmission resonance at a wavelength around 5 μm preserves a high signal-to-noise ratio down to an illuminated area of 10 × 10 μm^2^, which corresponds to less than 2 × 2 times the operation wavelength. The new degree of freedom along the vertical direction opens new possibilities in terms of spectral selection and hot-spot engineering^35^,^36^,^37^.

The manuscript is structured as follows. First we present the general structure of the coaxial array and the fabrication technique. Special attention is paid to the fabrication steps which allows defining the most important parameters of the array: the lattice period, the antenna length, and the size of the nanocoaxial apertures. Then we illustrate the theoretical and experimental IR spectra of the array, with particular emphasis on the spectral tunability and size reduction of each filtering element. More details on the numerical calculations, on the impact of the geometrical parameters of the array and on the parasitic absorption in the IR range are presented in the Supplementary Information.

## Results and Discussion

**Figure 1:**
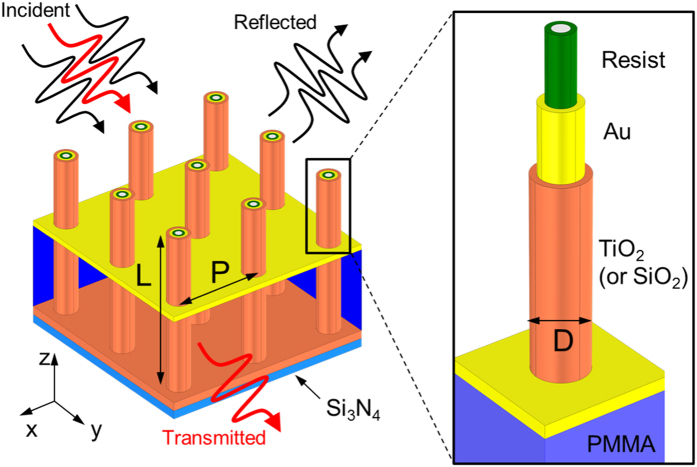
Sketch of the investigated array of coaxial antennas and detail of the radial multilayer structure (inset). The length of the antenna is denoted with L, the lattice period with P, and the total diameter of each antenna with D.

The system under investigation is sketched in Fig. 1. It consists of an array of 3D coaxial antennas with a multilayer structure along the radial direction. This is shown in detail in the inset of Fig. 1. Going from the inner to the outer shell, each antenna is made of a hollow cylinder of polymeric resist (green). A metallic layer (yellow) is deposited on this polymeric scaffold, and it is conformally coated with a thin dielectric layer (orange). A planar metallic layer cuts the array normally to the main axis of the antennas. The outer dielectric shell of each antenna defines a nanometric coaxial aperture which is the key element for the selective spectral transmission and for the strong electromagnetic field enhancement. The position of the metallic plane is defined by the thickness of the PMMA spacer layer (dark blue in Fig. 1). The light incident from the top excites a set of collective resonances, whose spectral positions are determined by the length of the antenna, by the lattice period, and by the diameter. The strong lateral scattering is provided by the out-of-plane geometry. The nanometric apertures with the central protruding antenna contribute to the spectral selectivity of our design. Light at specific wavelengths (red arrows in Fig. 1) can tunnel through the plane and be transmitted to the far-field. The other wavelengths (black arrows) are reflected back and rejected by the filter.

**Figure 2:**
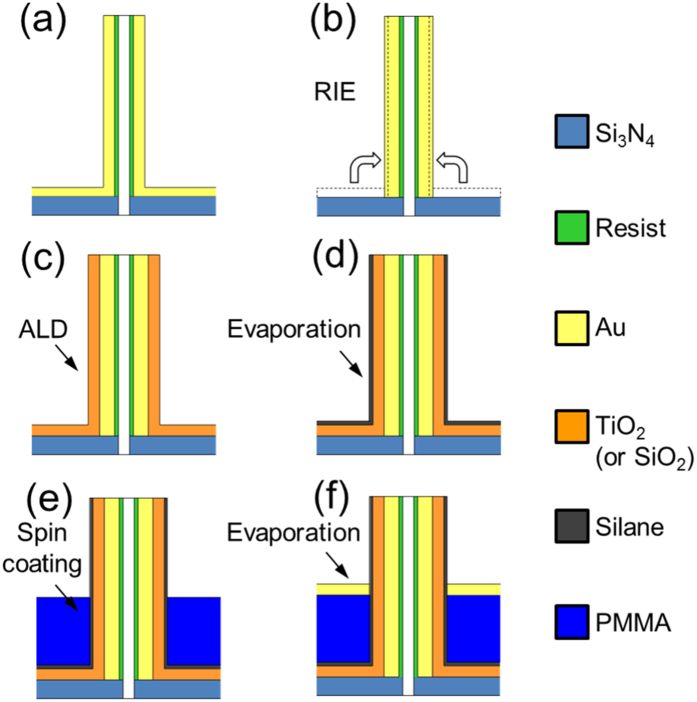
Schematic flow for the fabrication method. The starting template (**a**) is an array of hollow 3D antennas made of polymeric resist (green) obtained by FIB milling, which is coated with a thin gold (yellow) layer. (**b**) Reactive Ion Etching (RIE) stage to remove the gold deposited on the bottom Si_3_N_4_ membrane (light blue). (**c**) Atomic Layer Deposition (ALD) stage to deposit a conformal layer of TiO_2_ (or SiO_2_, orange). (**d**) Evaporation stage in presence of silane (dark grey) atmosphere. **(e)** Spin coating stage to deposit the PMMA (dark blue) layer. (**f**) Evaporation of a thin gold layer.

The main steps of the fabrication method are illustrated in Fig. 2. The starting template consists of an array of hollow 3D metallic antennas on a thin Si_3_N_4_ membrane (Fig. 2a). This template is obtained with several stages of Focused Ion Beam (FIB) milling and metal deposition. More details on the fabrication technique can be found in ref. 35 and in the Methods section. To enable the transmission of light through the sample, the gold deposited on the Si_3_N_4_ membrane is removed with a reactive ion etching (RIE) stage (Fig. 2b). As a consequence of RIE, a fraction of the removed gold tends to re-deposit on the antenna walls (dashed lines in Fig. 2b). The lateral gold thickness thus slightly increases (150–200 nm). The next step consists in the deposition of a thin (100–300 nm thick) conformal layer of TiO_2_ or SiO_2_ with Atomic Layer Deposition (ALD–Fig. 2c). This step concludes the radial multilayer structure of the antennas, and it is crucial to define the annular aperture which allows the flow of light to the other side of the sample.

In the next steps, we complete the design of the array in the out-of-plane direction (z). The first step (Fig. 2d) is a controlled silanization of the sample. The surface becomes oleophobic, and new materials can thus be deposited on the bottom plane without attaching to the lateral walls of the antennas^38^. A layer of PMMA is then spin-coated on the sample (Fig. 2e).

The thickness of this layer defines the height of the metallic plane which cuts the antennas normally to their main axis. The last fabrication step is the evaporation of a thin (80–100 nm) gold layer on the PMMA layer without rising on the wall of the antenna (Fig. 2f).

**Figure 3:**
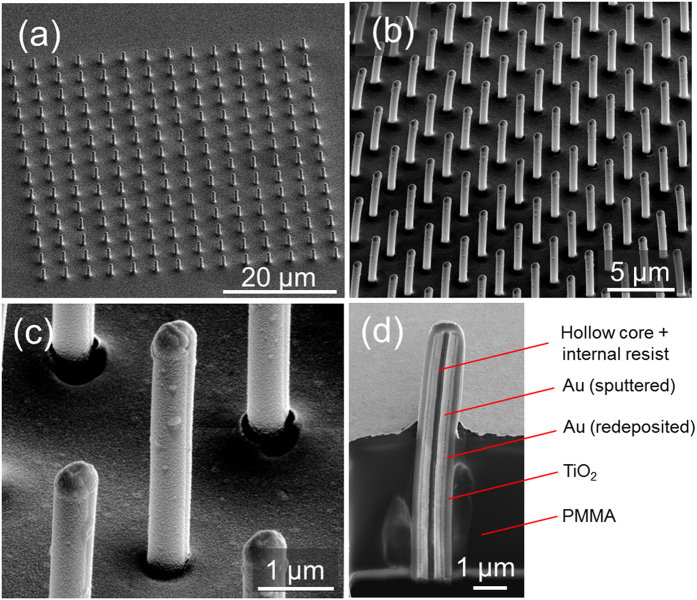
SEM images of a fabricated sample: (**a**) magnification 2500× (showing the entire area of the array, which is around 60 × 60 μm^2^), (**b**) magnification 6000×, (**c**) magnification 32500×. (**d**) Cross-sectional SEM image (magnification 17500×) of a single antenna showing its multilayer radial structure. The different materials are indicated.

The SEM images of fabricated samples are reported in Fig. 3. The whole array (Fig. 3a) has a footprint around 60 × 60 μm^2^. The typical lattice periods P and antenna lengths L are in the range 2–10 μm, and are both comparable with the operation wavelengths. The total diameter D of the antennas is in the range 400–700 nm.

A close view of a single antenna is reported in Fig. 3c, and an exemplificative cross section is shown in Fig. 3d. All the radial layers sketched in Fig. 2 are clearly evident in the cross section.

**Figure 4:**
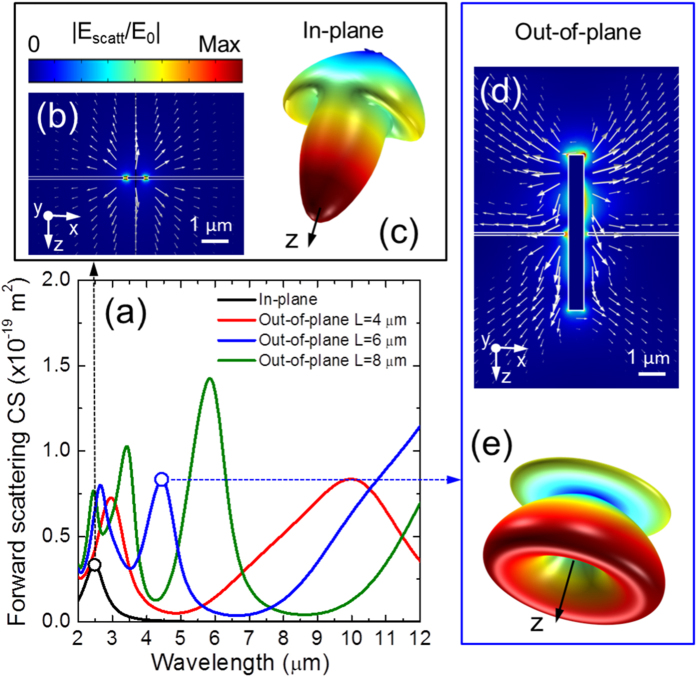
Comparison between in-plane and out-of-plane coaxial structures. (**a**) Theoretical forward scattering cross sections for a conventional 2D annular aperture (black line) and for three single coaxial antennas with L = 4 μm (red line), 6 μm (blue line) and 8 μm (green line). A TM-polarized plane wave with an incidence angle of 30° is assumed. Near- (**b**) and far-field (**c**) plots of the electric field scattered by an in-plane annular aperture at the resonant wavelength λ = 2.5 μm. The white arrows denote the Poynting vector associated to the scattered field. Near- (**d**) and far-field (**e**) plots of the electric field scattered by a single out-of-plane coaxial antenna with L = 6 μm at the wavelength of the TM 1 resonance, λ = 4.4 μm.

The scattering properties of a single coaxial antenna are investigated with finite-elements (FEM) calculations performed with the Comsol Multiphysics^®^ software^39^. More details are presented in the Supplementary Information. The results are presented in Fig. 4, where we compare the theoretical forward scattering cross sections (CS) of our new 3D out-of-plane design and of a conventional in-plane annular aperture^14^,^16^,^17^. To reproduce the FTIR experimental conditions, we assume a TM polarized plane wave incident at 30°. For this general comparison we assume that both the systems are made of gold and the surrounding medium is air. In both cases, the inner radius is equal to 300 nm, and the air gap is 150 nm.

The CS of the in-plane annular aperture (black line in Fig. 4a) shows a single resonance at λ ≈ 2.5 μm. Since this structure does not extend in the z-direction, the lateral scattering is weak. This is evident in the near field plot of Fig. 4b, where the Poynting vector of the scattered field (white arrows) is mainly directed along z. The far-field plot of Fig. 4c confirms this trend: the electric field has a peak intensity along the z-axis with relatively weak lateral lobes. The forward scattering CS for an out-of-plane coaxial antenna is substantially different. Three exemplificative spectra are reported in Fig. 4a for lengths of 4 μm (red line), 6 μm (blue line) and 8 μm (green line). Under TM incidence, several resonances appear: we denote the fundamental one as TM 0, and the higher-order ones as TM 1, TM 2, etc. The spectral position of each peak is determined by the length of the antenna. For the case L = 6 μm, the TM 0 resonance falls out of the plot range, while the TM 1 is located at λ ≈ 4.4 μm. Here we focus on the TM 1 resonance which can be clearly observed in the experimental FTIR spectra. The TM 0 resonance, instead, is strongly damped by the parasitic absorption in the dielectric materials. We report the corresponding near- and far-field plots in Fig. 4d,e, respectively. As it can be clearly seen, both the plots show a V-shaped profile with strong lateral lobes. From a comparison of the arrow plots in Fig. 4b,d, the angular distributions for the scattered field are very similar both in reflection and in transmission. Thanks to the extruded 3D geometry, the angular distribution of the coaxial antenna is substantially modified compared to a conventional annular aperture.

**Figure 5:**
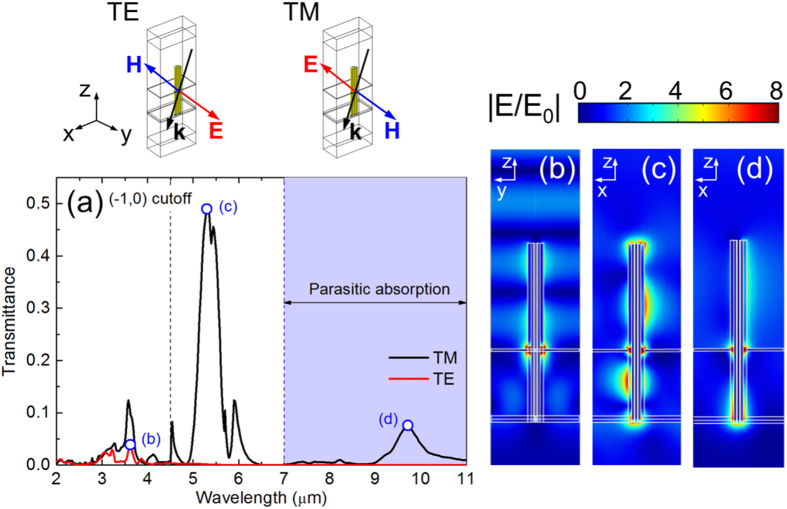
(**a**) Theoretical transmittance spectra for an array of coaxial antennas with P = 3 μm, L = 6 μm, D = 550 nm, and a coaxial gap of 100 nm. A plane wave is incident at 30°. The data for TE and TM polarizations are reported with red and black lines respectively. The main resonances are marked with blue open symbols. Near-field plots of the electric field enhancement |E/E_0_| in the unit cell for the main TE resonance (**b**, λ = 3.6 μm), longitudinal TM 1 resonance (**c**, λ = 5.3 μm), and longitudinal TM 0 resonance (**d**, λ = 9.7 μm). The range of parasitic absorption in the dielectric materials (λ longer than 7 μm) is delimited with a vertical blue dashed line. The wavelength of the lowest order diffraction cutoff is marked with a vertical black dashed line. The sketches of the incidence configurations for TE and TM polarizations are also reported.

For filtering applications, several coaxial antennas have to be packaged in an array configuration. In the theoretical analysis we assume a linearly polarized plane wave. The incidence conditions are sketched in Fig. 5. The electric field for the case of TE polarization is always orthogonal to the main axis of the antenna at any incidence angle. For this reason, only the weak *transverse* resonances of the antenna can be excited. The electric field for TM polarization, instead, has also a longitudinal component along the main axis of the antenna at tilted incidence. Both *transverse* and *longitudinal* modes can thus be excited. From the previous analysis of the isolated antenna, we expect that the purely longitudinal resonances will give a stronger lateral scattering. This boosts the collective effects in the array configuration, a situation which is particularly promising for filtering applications.

The theoretical transmission spectra for an array of antennas with P = 3 μm, L = 6 μm, and D = 550 nm are reported in Fig. 5a. A tilted incidence of 30° is assumed. Under TM polarization, the zero-order transmittance T_0_ shows two distinct peaks due to the longitudinal resonances. The TM 0 resonance is around λ = 9.7 μm, while the TM 1 peak is centered around λ = 5.3 μm. The TM 1 peak is strongly affected by the parasitic absorption in the PMMA (transmission dip at 5.8 μm). Several secondary peaks are present in the range between 3 and 5 μm. These are due to the transverse modes of the coaxial array.

The longitudinal TM 0 resonance is substantially blue-shifted compared to the single-antenna configuration (Fig. 4a, blue line), while the longitudinal TM 1 peak is slightly redshifted. The resonances are associated with intense hot-spots in the annular aperture and at the back of the array (Fig. 5c,d). The field enhancement |E/E_0_| in the gap reaches a maximum value around 30 for both the longitudinal resonances.

Under TE polarization, only a couple of small peaks are visible in transmission, in the same spectral range of the transverse TM 1 resonance (between 3 and 5 μm). Also in this case, a maximum field enhancement |E/E_0_| around 30 is obtained in the aperture (Fig. 5b). With a proper engineering, these hot-spots could be useful for biosensing^40^, second-harmonic generation^19^, and optical trapping^21^.

An important aspect for a spectral filter is the direction of propagation of the transmitted beam. The grating equation determines the cutoff for forward and backward diffraction in air:


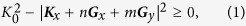


where K_0_ = 2π/λ_0_ denotes the amplitude of the incident wavevector, λ_0_ the wavelength in air, **K**_x_ = K_0_ sin(θ)**e**_x_ is the in-plane component of the incident wavevector, θ is the angle of incidence, **G**_x,y_ = 2π/P **e**_x,y_ are the basis vectors in the reciprocal lattice, and m and n are relative integers denoting the diffraction order of interest.

For the case of the lowest diffraction cutoff (n = −1, m = 0), Eq. (1) simply reduces to:


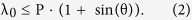


The fundamental diffraction cutoff is marked with a vertical dashed black line in Fig. 5a. Both the longitudinal TM 0 and TM 1 resonances fall below the diffraction cutoff, so the far-field transmission is in the same direction of incidence.

**Figure 6:**
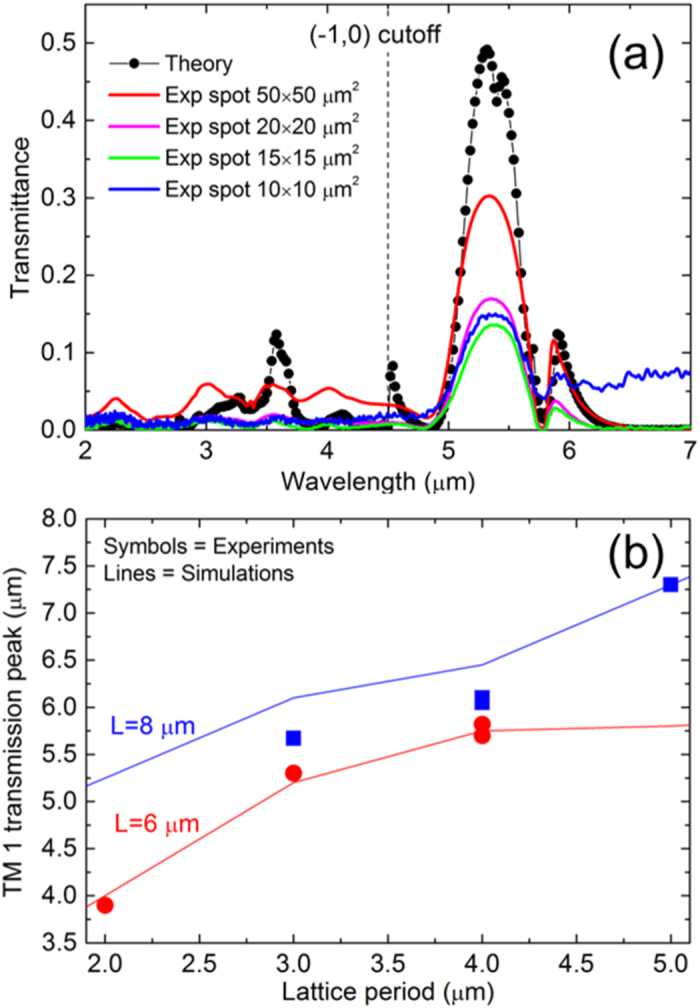
(**a**) Experimental (solid lines) and theoretical (black line and symbols) transmission spectra for an array of coaxial antennas with P = 3 μm, L = 6 μm, and D = 550 nm. The different experimental curves correspond to different spot sizes ranging from 10 × 10 to 50 × 50 μm^2^. (**b**) Tunability of the longitudinal TM 1 transmission peak. The experimental data for two sets of arrays with different lengths and lattice periods are reported with symbols. Red and blue symbols correspond to L = 6 and 8 μm, respectively. The same trends obtained with electromagnetic calculations are reported with lines.

The amplitude of the transmission peaks also depends on the incidence angle. In the Supplementary Information, we report the transmission spectra for the array investigated in Figs 5a and 6a. The maximum amplitude for the TM1 longitudinal peak is obtained in the range 30–45°. We remark that oblique incidence is a necessary condition to excite the longitudinal resonances which leads to the strongest transmission peaks. For this reason,in view of a future application, the proposed coaxial unit should be mounted in a tilted configuration.

Multi-spectral imaging with high spatial resolution requires reducing the size of the array down to a few times the operation wavelength. In this way, micro-structured pixels can select and analyze different wavelengths and be densely packaged in a CCD architecture. To investigate this aspect, we fabricated several arrays of coaxial antennas with different lengths and lattice periods. The samples are characterized by means of FTIR spectroscopic measurements (details in the Methods section). Our FTIR setup comprises two parabolic mirrors mounted in Cassegrain configuration to focus the incident beam and collect the transmitted light. For this reason all the measurements are performed at a fixed incidence of 30°. The measured IR transmission spectra of an array with L = 6 μm, P = 3 μm and D = 550 nm are reported in Fig. 6a with solid lines. The lateral footprint of the array is 60 × 60 μm^2^, and we modify the illumination area by changing the aperture of the slits in our FTIR setup.

The size of incident squared beam is in the range 10 × 10–50 × 50 μm^2^. The theoretical results obtained with FEM electromagnetic calculations are also reported with a line and symbols. The array shows the main longitudinal TM 1 peak at λ = 5.3 μm plus a set of transverse resonances at shorter wavelengths. The fundamental TM 0 resonance falls out of the reported range and it is strongly damped by the parasitic absorption in PMMA, TiO_2_ and Si_3_N_4_ (details in the Supplementary Information). The measured height of the main peak at 5.3 μm is lower than the calculated value, and this is probably due to fabrication imperfections, especially in the region surrounding the aperture. The remaining difference at shorter wavelengths can be attributed to the different focalization conditions adopted in the simulations and in the experiments. Thanks to the strong lateral scattering of light of the longitudinal TM 1 mode, the collective effects are present even if just a few unit cells are illuminated by the incident beam. The resonance has a high signal-to-noise ratio down to an illuminated area of 10 × 10 μm^2^, which is less than 2 × 2 times the operation wavelength. This values compares favorably with existing micro-filters^41^,^42^,^43^,^44^, and it is promising for the application of our novel coaxial structures in the field of multi-spectral imaging. Terahertz spectroscopy and security applications are other potential fields of application for this new out-of-plane infrared plasmonic structure.

The tunability of the transmission resonance is a crucial aspect for the application of the proposed 3D coaxial array as an IR filter. This aspect is investigated theoretically and experimentally, and the results are reported in Fig. 6b. We fabricated two sets of samples with antenna lengths of 6 and 8 μm and different lattice periods in the range 2–5 μm. An annular aperture of 150 nm is defined by means of the outer TiO_2_ shell. The peak associated with the TM 1 resonance is mapped for different lattice periods: the experimental results are reported with symbols. The trends obtained with electromagnetic calculations are reported with lines. The TM 1 peak can be tuned in the mid-infrared range by changing the lattice period, and a good agreement is found between theoretical and experimental results. Small variations of the experimental points around the theoretical trends are evident for the case L = 8 μm. These are due to small variations in the antenna length around the nominal values.

## Conclusions

We designed, fabricated and investigated a new configuration of 3D coaxial metallic antennas working in the infrared. The structures are based on a hollow metallic core with an outer dielectric shell. A novel and flexible fabrication technique has been developed in order to control the radial structure of the antenna as well as the vertical design. Thanks to the strong in-plane scattering provided by the 3D geometry and to the presence of the nanometric coaxial gap, the arrays are characterized by a selective spectral transmission and high field enhancement. The transmission resonances have been investigated both theoretically with FEM electromagnetic calculations, and experimentally with FTIR spectroscopic measurements. In view of an application in the field of IR filtering, we demonstrate the tunability of the transmission resonances by changing the lattice period and/or the antenna length. We also demonstrated that the arrays preserve their spectral selectivity down to an illuminated area of 10 × 10 μm^2^, which correspond to less than 2 × 2 times the illumination wavelength. This is a promising result for the future application of our 3D coaxial design in a CCD architecture for multi-spectral imaging with high spatial resolution.

## Methods

### Fabrication of the 3D array of coaxial antennas

Our new fabrication method enables the production of arrays of three-dimensional coaxial antennas embedded in a PMMA layer. We combine nanoscale precision with fast processing and large area fabrication capabilities. The starting template (Fig. 2a) consists of an array of 3D hollow metallic antennas. This scaffold is obtained by means of secondary electron lithography generated by ion beam milling.

A polymer, resist Shipley S1813^45^, is spin-coated on a thin (100 nm) silicon nitride membrane. The thickness of the resist depends on the spinning velocity and it is highly controllable. The thickness can vary from a few tens of nanometers up to tens of microns. In order to guarantee a high conductivity of the sample, a few nanometers of gold are sputtered on the back side of the membrane. Focused Ion Beam (FIB) is used to achieve the milling process, drilling through the membrane from the backside (FEI NanoLab 600 dual beam system^46^). The same instrument is also used to acquire the SEM images reported in the main text. The Gallium ions of the beam interact with the resist polymer producing low-energy secondary electrons which break the carbon-carbon bonding in the region close to the milled surface. The exposed resist passes from a positive tone to a negative tone, becoming insoluble. Subsequently, the gold layer on the back side of the membrane is removed with a wet etching using a standard gold etchant. Than the sample is immersed in a acetone to remove the unexposed resist. The exposed resist becomes the scaffold of the final structure. The lateral footprint of the fabricated arrays is 60 × 60 μm^2^. An oxygen plasma etching is performed in order to reduce the thickness of the scaffold and to decrease the lateral roughness. The antennas are then covered with a thin film of a noble metal (gold, 100 nm thick) by means of sputter coating. The whole sample surface is thus covered with an continuous conductive film whose shape is determined by the underlying scaffold of the antennas. The gold from the substrate is removed to enable the transmission of the light through the sample. The removal is performed with Reactive Ion Etching (RIE) stage (Sentech SI 500 ICP-RIE plasma etcher^47^), applying highly powerful Argon plasma. This kind of milling is extremely vertical so the gold is removed from the substrate but is left untouched on the antennas. A small amount of gold redeposits on the antennas walls increasing the gold thickness to around 150–200 nm. A conformal layer (thickness 100–300 nm) of TiO_2_ or SiO_2_ is deposited with an Atomic Layer Deposition (ALD) machine (Oxford Instrument FlexAl Atomic Layer Deposition System^48^). This machine guarantees precise thickness and isotropic deposition. At this point the radial design of the antenna is complete. The next steps define the out-of-plane structure of the array.

The sample is coated with trichloro-perfluorooctyl-silane in order to obtain an oleophobic surface. The silanization is performed with a vacuum chamber; the sample is inserted in the chamber with few microliter of silane. A rough vacuum is generated into the chamber and the sample is left inside 1 hour. The sample is thus coated with a few layers of silane. At this point a layer of PMMA 950 A11 is spun to cover approximately half of the antennas height. As the last step, we perform an evaporation of 80–100 nm of gold with an electron beam evaporator (Kenosistek E-beam evaporator^49^). The evaporation is performed to achieve an extreme vertical coating: with this method the gold does not deposit on the vertical walls of the antennas.

An important point regarding our new fabrication technique is its flexibility. The milling process allows producing an array of antennas with different dimension, pitch and height. The thickness of the gold layer deposited on the antenna as well as the size of the coaxial apertures can be controlled with high precision. The thickness of the PMMA layer, and hence the height of the gold mid-plane can also be tuned easily during the spin-coating process.

### FTIR spectroscopy measurements

The infrared transmission spectra of the samples are measured with a Thermo Scientific Nicolet IS50 Fourier transform micro spectrophotometer^50^ (coupled with a Thermo scientific Nicolet continuum FT-IR microscope) in the range between 650 cm^−1^ and 7000 cm^−1^ (wavelength between 1.5 μm and 15 μm). The instrument is equipped with a tungsten filament heated at 1200 K, which serves as the light source. The incident beam is split by means of a KBr beam splitter. The transmitted beam is focused on the sample, while the reflected beam is directed into one arm of a Michealson interferometer. A mirror is placed at the end of this arm, and it is moved periodically by means of a motor. For each position, only one wavelength of the incident light is transmitted to the photodetector. A full interferogram can thus be collected every time the mirror completes a round trip motion. The interferogram is then converted into a frequency spectrum with a computer software. Each round trip of the mirror represent a single scan of the spectrum. The resulting transmission spectrum is an average over 200 scans. The excited area is selected by means of a modifiable slit aperture through two Cassegrain objectives (0.58 numeric aperture, 15× magnification, 30 degrees fixed incidence angle). The first objective is used to focus the light incident on the sample, while the second one is used to collect the transmitted light. Due to the arrangement of the internal optics, the instrument can only perform measurements at an incidence of 30°. A reference spectrum is collected before each acquisition. The aperture of the slits is in the range 10 × 10 μm^2^–50 × 50 μm^2^. For the smallest aperture (10 × 10 μm^2^) the spectrum is averaged over 800 scans to reduce the noise.

## Additional Information

**How to cite this article**: Jacassi, A. *et al*. 3D coaxial out-of-plane metallic antennas for filtering and multi-spectral imaging in the infrared range. *Sci. Rep*. **6**, 28738; doi: 10.1038/srep28738 (2016).

## Supplementary Material

Supplementary Information
