# Supplementary material for: 3D coaxial out-of-plane metallic antennas for filtering and multi-spectral imaging in the infrared range
Source: Sci Rep. 2016 Jun 27;6:28738. doi: 10.1038/srep28738 (PMC4921826; doi:10.1038/srep28738)
Supplement: Supplementary Information [file srep28738-s1.pdf]

Supplementary Information

Title: 3D coaxial out-of-plane metallic antennas for filtering and multi-spectral imaging in the infrared range

Andrea Jacassi[1,2,‡], Angelo Bozzola[1,‡], Pierfrancesco Zilio[1], Francesco Tantussi[1], and Francesco De Angelis[1]*

[1] Istituto Italiano di Tecnologia – Via Morego, 30, I-16163 Genova, Italy

[2] Università degli Studi di Genova, via Balbi, 5, I-16126, Genova, Italy

[*] Corresponding author's email: francesco.deangelis@iit.it

[‡] A. J. and A. B. equally contributed to this work.

SI#1: Details on the electromagnetic calculations

All the electromagnetic calculations have been carried out using the commercial software Comsol Multiphysics® (RF module). The refractive index of gold was taken from Ref. 1; the values for PMMA from Ref. 2, and those for TiO_2, SiO_2 and Si_3N_4 from Ref. 3.

For the calculations regarding single in-plane and out-of-plane antennas (Fig. 4 of the main text), we assumed a TM polarized plane wave with an angle of incidence of 30°. The simulation domain is enclosed within perfectly matched layers (PML) in order to avoid spurious reflections from the boundaries of the simulation box. The forward scattering cross sections (Fig. 4a of the main text) are obtained by calculating the flux of the scattered Poynting vector over a surface enclosing the bottom part of the antenna. The surface is determined by the bottom boundaries of the simulation box and by the metallic mid-plane.

For the calculations regarding the arrays of antennas, the electromagnetic field is calculated over half of the unit cell for symmetry reasons. A linearly polarized plane wave with an angle of incidence of 30° is assumed, and both the TE and TM polarizations are considered. The incidence configurations are sketched in Fig. 5 in the main text. In correspondence of the longer side of the simulation box the lateral PML domains were replaced with Bloch-Floquet periodic boundary conditions. On the shorter side of the simulation box, the PML domains were replaced with Perfect Electric Conductor (PEC) surfaces for TE incidence, and with Perfect Magnetic Conductor (PMC) surfaces for TM incidence. PML layers are assumed at the top and bottom boundaries of the unit cell.

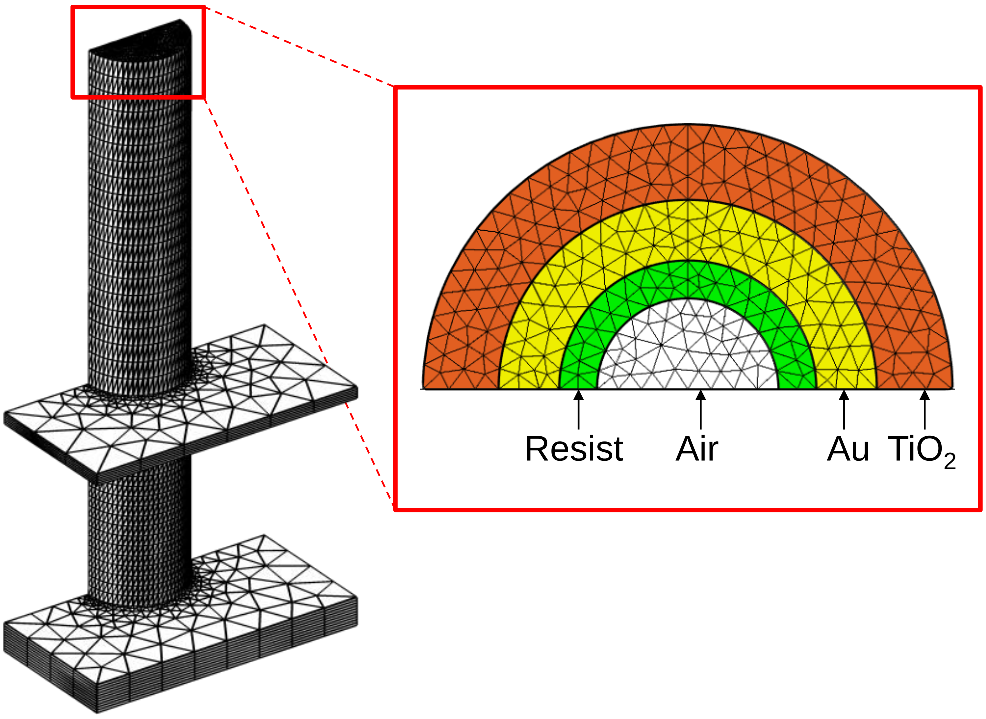

Fig. SI1 (Color online): Example of the numerical mesh used in the simulation of the 3D arrays of coaxial antennas. Inset: the triangular mesh used for the multilayer radial structure of each antenna.

Compared to the configuration adopted in the FTIR measurements, a plane wave is a good approximation of the incident focused beam at its focal plane. The presence of the focusing objective determines a spread in the angular distribution of the incident field. From FEM calculations (not shown), this spread induces a change in the absolute height of the peaks and in their width. While these effects might be significant for the transverse resonances at high energies, they do not substantially change the shape of the main longitudinal peaks. The full calculation of the scattered field taking into account the exact shape of the incident wave front would require a very large supercell, and this is out of our computational resources.

An exemplificative plot of the simulation mesh used in the antenna domains is reported in **Fig. SI1**. The top of the antenna (detail in the inset) is meshed with a triangular mesh. The maximum mesh size is tuned in order to allocate at least three mesh nodes within each layer. Along the vertical direction, the mesh is swept at constants steps equal to $\lambda/10$, where λ denotes the shortest wavelength in the simulation (typically λ=2 μm). The same method is used to mesh the planar domains (metallic mid-plane, TiO_2 and Si_3N_4 membrane). In this case, the triangular mesh is swept along the vertical direction with a distribution of five elements. The air and PMMA domains are meshed with a free tetrahedral mesh with

maximum cell size of λ/5 and λ/(5×n_{PMMA}), respectively (here n_{PMMA} denotes the average real part of the refractive index of PMMA in the IR, which is approximately equal to 1.55).

With these settings, the unit cell of the array is meshed with a total of 20000 – 80000 mesh elements, depending on the length of the antenna and on the lattice period. The calculation of each IR spectrum in the range 2-12 μm (wavelength step of 20-40 nm) takes between 2 and 10 hours on a modern workstation equipped with two Intel Xeon processors (3.1 GHz – 8 cores each) and 256 Gb of RAM memory.

SI#2: Parasitic absorption in the IR range

Our new configuration of 3D coaxial antennas involve several metallic and dielectric materials. In order to maximize the amplitude of the transmission resonances, the parasitic absorption has to be minimized with a proper choice of the materials. The impact of the parasitic absorption is illustrated in **Fig. SI2**, where we report the theoretical spectra for the array of coaxial antennas with L=6 μm, P=3 μm, and D=550 nm (the same sample investigated in Fig. 5 and 6 of the main text). As it is clear from the plot of Fig. SI2a, only a fraction of the incident light is transmitted to the far-field (red line), while the rest is either absorbed (black line) or back reflected (blue line).

The total absorption spectrum of Fig. SI2a is decomposed into the absorption spectra in the different materials involved in the coaxial antennas. This is done by spatially integrating the wavelength-dependent ohmic losses over the different domains: the result is presented in Fig SI2b. Gold is the most important absorber material in the investigated range (green line in Fig. SI2b). When a resonance is excited, a fraction of the incident power is always dissipated in the metallic walls of the antenna or in the mid-plane. PMMA presents a set of sharp vibrational bands all over the IR range, with a prominent absorption peak at λ=5.8 μm (red line of Fig. SI2b). This peak is responsible for the minimum in the transmission spectrum reported in Fig. SI2a and in Figs. 5a and 6a of the main text. TiO_2 has been chosen as the dielectric for the outer shell. This is a critical position. In fact, when the system is driven at resonance, strong field enhancements are present in the TiO2 shell, which is in direct contact with the metallic walls of the antenna. TiO_2 absorbs a substantial fraction of the incident power at wavelengths longer than 9 μm. Si_3N_4 has similar absorption properties in the same spectral range. These two materials are responsible for the strong damping of the fundamental TM 0 resonance, which is barely visible in the transmission spectrum at λ=9.7 μm (Fig. SI2a).

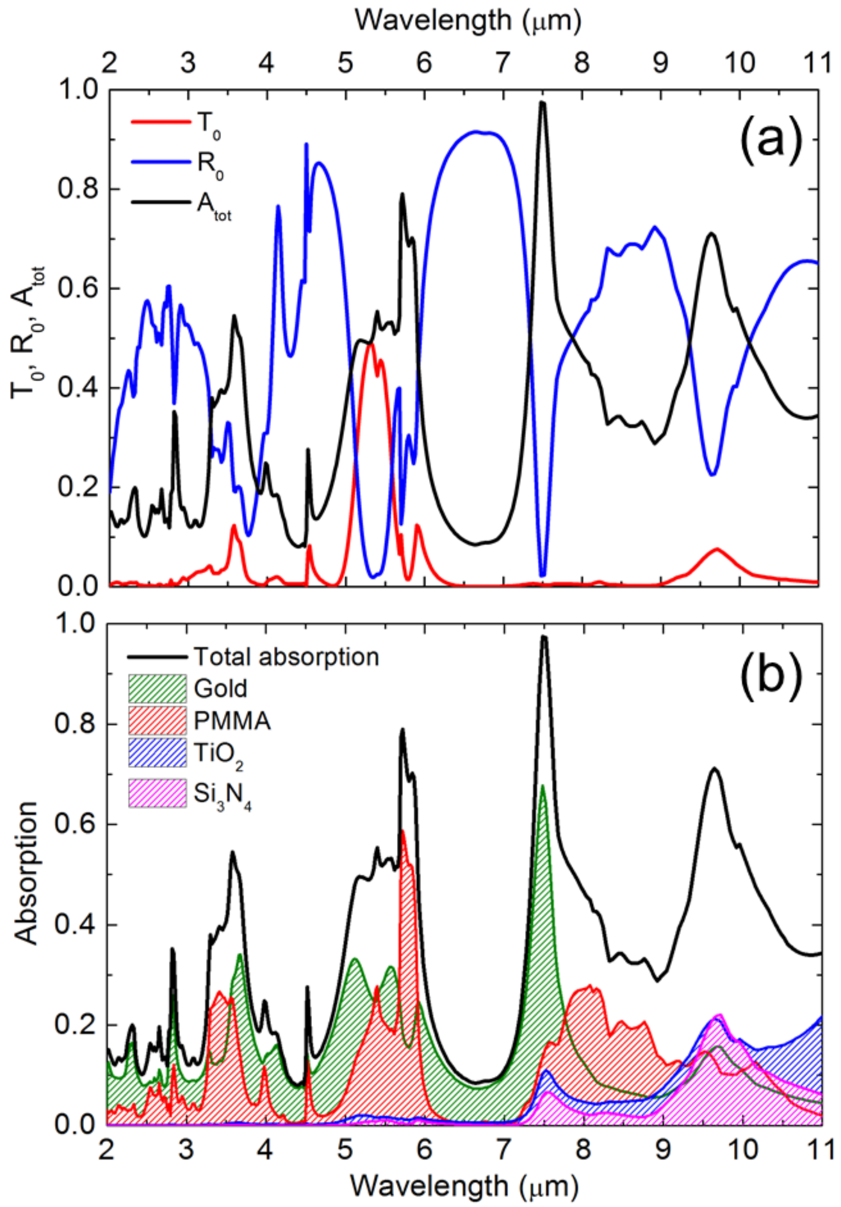

Fig. SI2: (Color online) Impact of the parasitic absorption for an array of 3D coaxial antennas with L=6 μm, P=3 μm, and D=550 nm illuminated by a TM-polarized plane wave at an incidence of 30°. **(a)** Theoretical transmittance (red line), reflectance (blue line) and total absorption (black line) spectra. **(b)** Contributions to the total absorption (thick black line) by the different materials involved in the array: absorption in gold (green line), in PMMA (red line), in TiO_2 (blue line) and in Si_3N_4 (magenta line).

SI#3: Angular dependence of the IR spectra

The angular dependence of the IR spectra for the array of coaxial antennas with P=3 μm, L=6 μm and D=550 nm is investigated by means of electromagnetic calculations for an

angle of incidence in the range 0-60°. The results for TM and TE polarizations are illustrated in **Fig. SI3a** and **SI3b**, respectively. Due to the different orientations of the electric field (inset of Fig. 5 in the main text), the two polarizations can excite two distinct sets of *transverse* and *longitudinal* resonances.

At normal incidence (θ=0°), the IR spectra for TE and TM polarizations coincide (black curves in Figs. SI3a and SI3b). In this configuration, only the transverse resonances of the array can be excited. These produce small peaks in the transmission spectra (amplitude of just 7%), and they are thus not interesting for filtering applications.

At oblique incidence (θ>0°) the electric field for TM-polarized light has also a component parallel to the axis of the antenna. For this reason, both *transverse* and *longitudinal* resonances can be excited. Their angular dispersion is illustrated in Fig. SI3a. The excitation efficiency of the TM0 mode around 9.7 μm (which is purely longitudinal) increases by increasing the incidence angle. However, due to the parasitic absorption, the maximum (theoretical) amplitude of this peak is just 16% at 60 degrees. In addition, the TM0 mode has a larger natural width compared to the longitudinal TM1 mode. These two facts make the TM0 mode less interesting for filtering applications. The excitation efficiency of the longitudinal TM1 mode around 5.3 μm is maximum between 30 and 45 degrees, with a theoretical transmission around 50%. For this reason, this is the most interesting resonance for filtering application. The longitudinal TM1 mode shows also a moderate redshift by increasing the angle of incidence (Fig. SI3a). The transverse TM1 peaks between 4 and 5 μm are excited by the in-plane component of the electric field (inset of Fig. 5 of the main text), and they produce shallow peaks in the transmission spectra.

TE-polarized light has the electric field polarized in the x-y plane of the array (inset of Fig. 5 in the main text). For this reason, it can only excite the *transverse* modes of the antenna, which are qualitatively analogous to the aforementioned transverse TM1 modes. Also in this case, the peaks in the transmission spectra (Fig. SI3b) are very small (less than 4% at θ>0°).

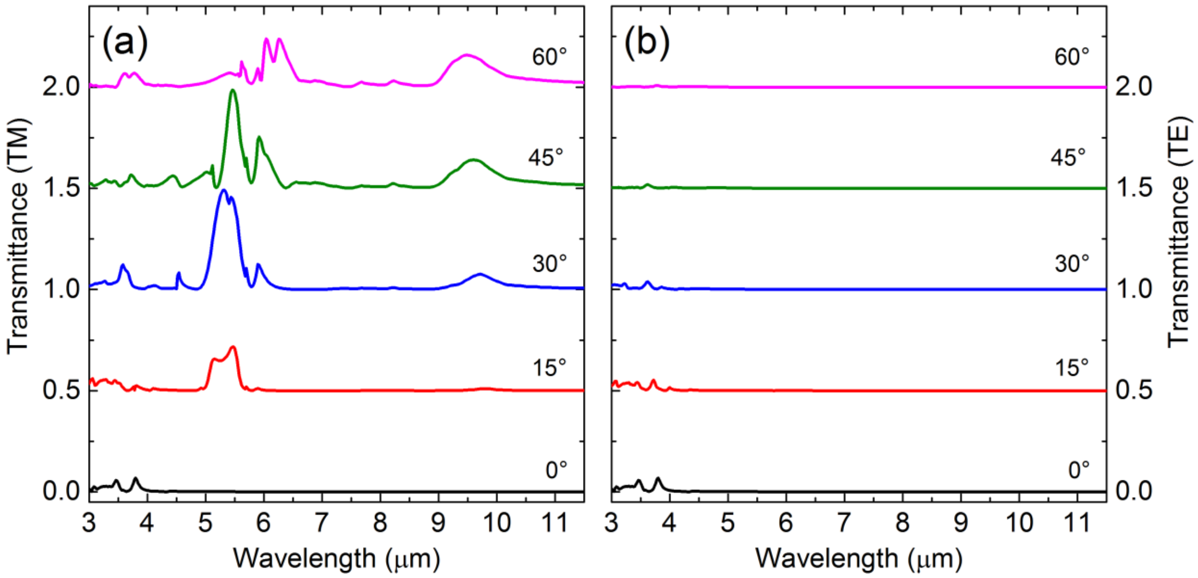

Fig. SI3: (Color online) Angular dependence of the IR spectra for an array of 3D coaxial antennas with L=6 μm, P=3 μm, and D=550 nm illuminated by a TM-polarized plane wave **(a)** and by a TE-polarized plane wave **(b)** at an incidence of 30°. All the spectra are vertically shifted by +0.5 for clarity.

References

1. Rakic, a D., Djurisic, a B., Elazar, J. M. & Majewski, M. L. Optical properties of metallic films for vertical-cavity optoelectronic devices. *Appl. Opt.* **37,** 5271–5283 (1998).

2. Jitian, S., Bratu, I. & Lazar, M. D. Determination of optical constants of polymethyl methacrylate films from IR reflection-absorption spectra. 26–29 (2012). doi:10.1063/1.3681958

3. Kischkat, J. *et al.* Mid-infrared optical properties of thin films of aluminum oxide, titanium dioxide, silicon dioxide, aluminum nitride, and silicon nitride. *Appl. Opt.* **51,** 6789–6798 (2012).
